# Targeted radionuclide therapy and diagnostic imaging of SSTR positive neuroendocrine tumors: a clinical update in the new decade

**DOI:** 10.3389/fnume.2025.1655419

**Published:** 2025-08-07

**Authors:** Katherine N. Haugh, Alexis M. Sanwick, Ivis F. Chaple

**Affiliations:** Department of Nuclear Engineering, University of Tennessee, Knoxville, TN, United States

**Keywords:** neuroendocrine tumors, radiopharmaceuticals, peptide receptor radionuclide therapy, theragnostics, diagnostic imaging

## Abstract

Neuroendocrine tumors (NETs) are a heterogeneous group of neoplasms characterized by their overexpression of somatostatin receptors (SSTRs), which can be utilized for peptide receptor radionuclide therapy. This review provides a comprehensive update on the clinical trials of radiolabeled SSTR-targeting radiopharmaceuticals since 2020, with a focus on somatostatin receptor agonists and antagonists radiolabeled with ^68^Ga, ^18^F, ^99m^Tc, ^177^Lu, ^161^Tb, ^212^Pb, ^67^Cu, and ^225^Ac. Head-to-head clinical trials demonstrate that radiolabeled SSTR antagonists such as [^68^Ga]Ga-DOTA-JR11 and [^68^Ga]Ga-DOTA-LM3 offer improved lesion detection and tumor-to-background ratios (particularly in liver metastases) compared to radiolabeled agonists like [^68^Ga]Ga-DOTA-TOC and [^64^Cu]Cu-DOTA-TATE. Additionally, ^18^F-labeled agents offer logistical and dosimetric advantages over ^68^Ga, due to ^18^F's longer half-life and cyclotron production, allowing for delayed imaging and increased availability to a wider range of patients. Emerging targeted alpha therapy agents, including [^225^Ac]Ac-DOTA-TATE, show promising results in treating disease resistant to conventional therapies due to the high linear energy transfer of alpha particles, which leads to improved localized cytotoxicity. Collectively, these developments support a shift toward more precise, receptor-specific theragnostics, emphasizing the need for further head-to-head clinical trials and integration of dosimetry-driven, personalized treatment planning in the management of NETs.

## 1 Introduction

Neuroendocrine tumors (NET) are heterogeneous tumors derived from the tissues associated with the embryonic neural crest, neuroectoderm, and endoderm (1). NETs form either diffusely or in organized cell clusters due to tumorigenic mutation events in hormonally programmed neuroendocrine precursor cells (2). Due to the variability of cell tissue mutations, NETs form throughout the body but are primarily found in the gastroenteropancreatic system (3). NETs commonly form well-differentiated tumors with a low proliferation rate and are categorized based on the extent of differentiation. NETs are classified as grades one through three, where grades one and two are well-differentiated tumors, while grade three is poorly differentiated. Table 1 compares the proliferation rates between NET grades according to the World Health Organization classification system.

**Table 1:** Grading system for NETs (72, 73).

Grade	Proliferation Rate (Lungs and thymus NETs)	Proliferation Rate (Gastroenteropancreatic NETs)
Grade 1	<2 mitoses per 10 high-power fields with no necrosis	<3% Ki67 index
Grade 2	2–10 mitoses per 10 high-power fields or a foci of necrosis	3%–20% Ki67 index
Grade 3	>10 mitoses per 10 high-power fields	>20% Ki67 index

In 2023, Wu et al. utilized the SEER database, finding that the average survival rate and incidence rate of NETs are increasing due to improved early diagnosis and novel therapeutic methods (4). The annual age-adjusted rate of NET occurrence per 100,000 people increased from 4.90 in 2000 to 8.19 in 2018. Advancements in diagnosis methods, biotherapy, chemotherapy, and nuclear medicine have extended the average relative survival rate of patients. While the overall 5-year NET survival rate is approximately 68.4%, the relative survival rate (RSR) varies by tumor site, with the rectum having the highest RSR and the lungs having the lowest RSR (5). For NETs located in the lungs, the median overall survival is 10 months (6). With the progressive increase in survival rate, pancreatic NET survival has increased from 58.90% in 2013 to 59.94% in 2018 (6). Since NETs demonstrate significant variability in tumor location and symptoms, they have historically gone undiagnosed at the initial stages (4). Most NETs are characterized by an overexpression of somatostatin receptors, which have been leveraged to develop targeted diagnostic imaging and radionuclide therapy agents.

**Figure 1:**
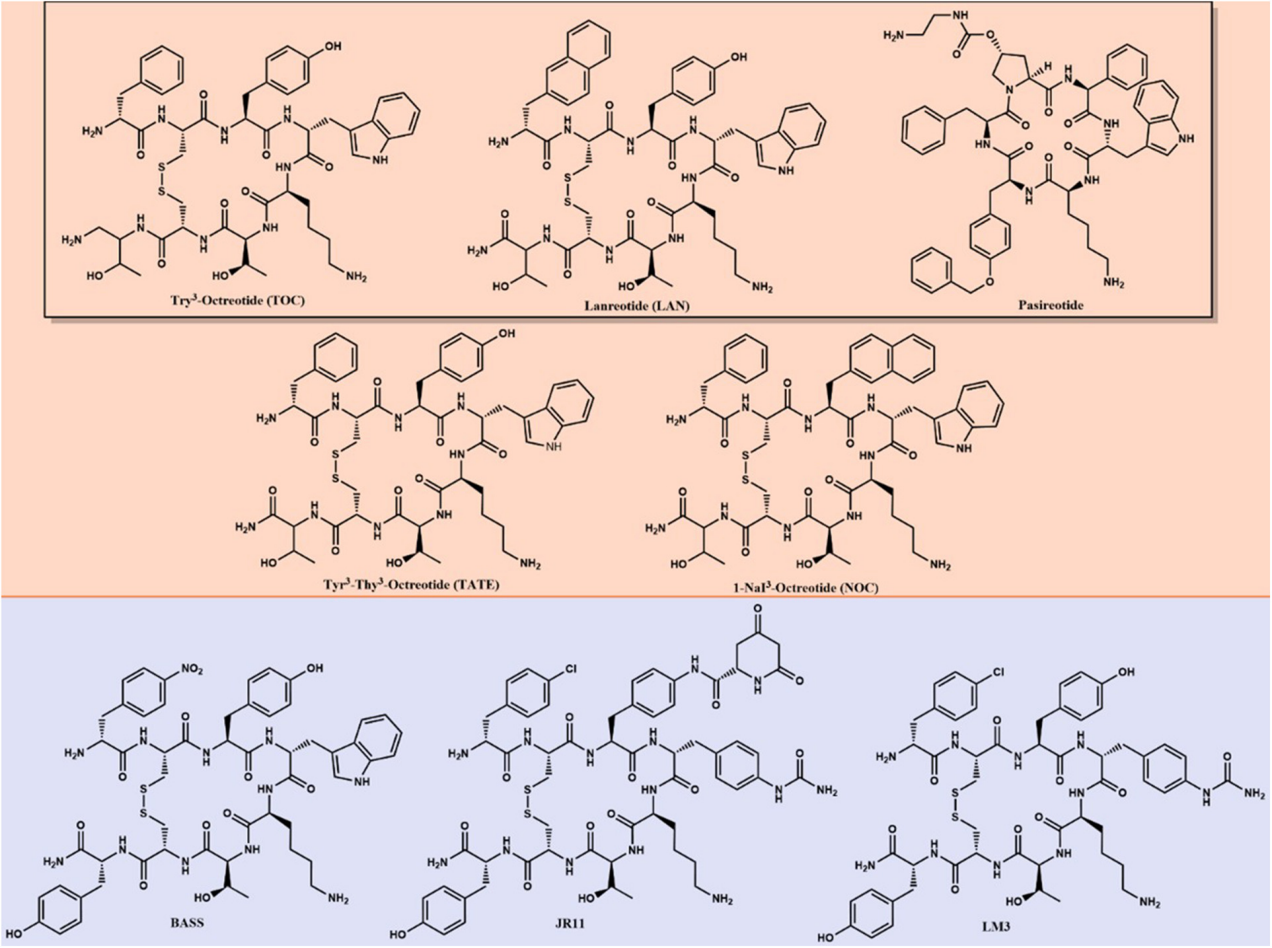
Long-lived SSAs—agonists (orange) and antagonists (purple)—are used as targeting agents for imaging and therapy of NETs. The black box surrounds FDA-approved SSAs for biotherapy.

Peptide Receptor Radionuclide Therapy (PRRT) has garnered continued interest as a secondary treatment option for somatostatin receptor (SSTR)-positive NETs (7). Since 1994, five radiopharmaceuticals have been FDA-approved for treating or diagnosing NETs that utilize somatostatin analogs (SSA). Figure 1 shows the different chemical structures of SSA differentiating between agonists and antagonists. [^111^In]In-pentetreotide (Octreoscan) was the first FDA-approved radiopharmaceutical for scintigraphy and SPECT imaging of grade one and two NETs in 1994 (8). [^111^In]In-pentetreotide was the gold standard of diagnostic imaging of NETs until kit preparation for [^68^Ga]Ga-DOTA-TATE (NETSPOT) was approved by the FDA in 2016 (9). In 2018, [^177^Lu]Lu-DOTA-TATE (Lutathera) became the first and only FDA-approved radiopharmaceutical for the treatment of NETs (10). A second ^68^Ga-labeled radiopharmaceutical, [^68^Ga]Ga-DOTA-TOC, was approved by the FDA in 2019 with a different SSTR specificity than [^68^Ga]Ga-DOTA-TATE (11). The most recent FDA-approved radiopharmaceutical for NET imaging is [^64^Cu]Cu-DOTA-TATE (Detectnet), which was FDA-approved in 2020 (12). Several clinical trials are currently underway to develop and approve diagnostic and treatment options for patients with NETs, which will be discussed in this review.

## 2 Diagnostic imaging agents

### 2.1 Advancement in galium-68 (^68^Ga) imaging

#### 2.1.1 [^68^Ga]Ga-DATA-TOC

DATA (6-amino-1,4-diazepine-triacetate) has been proposed as an alternative chelator to DOTA due to its high radiochemical yield that does not require heating to 95°C. Yadav et al. conducted a clinical trial involving 55 patients to compare the diagnostic efficacy of [^68^Ga]Ga-DATA-TOC with that of [^68^Ga]Ga-DOTA-NOC (13). The study found 98.6% concordance between [^68^Ga]Ga-DATA-TOC and [^68^Ga]Ga-DOTA-NOC for lesion detection, making [^68^Ga]Ga-DATA-TOC comparable to [^68^Ga]Ga-DOTA-NOC in terms of imaging quality, while also improving synthesis methods.

#### 2.1.2 [^68^Ga]Ga-DOTA-JR11

The first clinical trials using [^68^Ga]Ga-DOTA-JR11 were completed by Krebs et al. in 2019 to determine biodistribution, rate of tumor uptake, and absorbed dose in 20 patients (14). Using [^68^Ga]Ga-DOTA-JR11, positive lesions were detected in all 20 patients with an SUV_max_ of 13 in tumor regions of interest. Biodistribution was determined throughout the body, with low uptake levels occurring in the pituitary gland, parotid glands, salivary glands, thyroid, spleen, adrenal glands, and liver. The total effective dose for the patients was determined at 0.022 ± 0.003 mSv/MBq.

**Figure 2:**
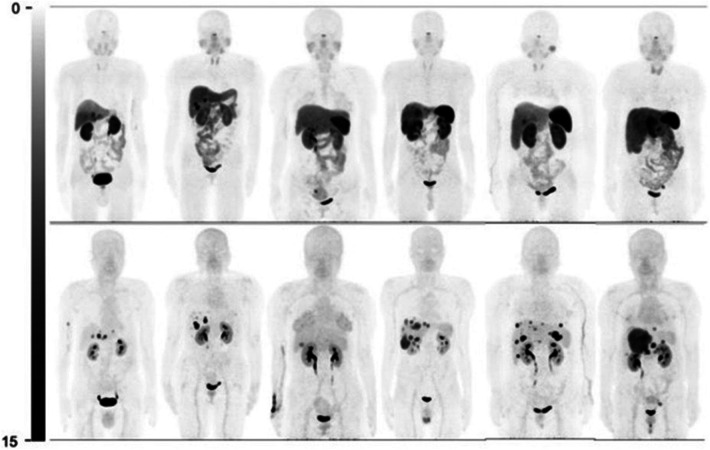
Comparison of [^68^Ga]Ga-DOTA-TATE (top) and [^68^Ga]Ga-DOTA-JR11 (bottom) biodistribution and tumor detection in 6 patients (15).

In 2020, Zhu et al. completed a head-to-head study comparing [^68^Ga]Ga-DOTA-JR11 to [^68^Ga]Ga-DOTA-TATE biodistribution with 31 enrolled patients (15). [^68^Ga]Ga-DOTA-JR11 and [^68^Ga]Ga-DOTA-TATE found 835 vs. 875 metastases, respectively. While [^68^Ga]Ga-DOTA-JR11 showed an overall lower tumor uptake, it detected 552 liver metastases, compared to [^68^Ga]Ga-DOTA-TATE, which detected only 365 lesions. [^68^Ga]Ga-DOTA-TATE detected more bone lesions than [^68^Ga]Ga-DOTA-JR11, with 158 and 88 metastases detected, respectively. Comparable results were obtained for lymph nodes and primary tumors. With the decreased biodistribution of [^68^Ga]Ga-DOTA-JR11, a better tumor-to-background ratio (TBR) was observed in the liver, resulting in improved image quality and tumor differentiation. The better TBR for [^68^Ga]Ga-DOTA-JR11 can be seen in Figure 2 where more lesions are visible using [^68^Ga]Ga-DOTA-JR11 than [^68^Ga]Ga-DOTA-TATE. Based on the study results, [^68^Ga]Ga-DOTA-JR11 may be an alternative imaging agent to [^68^Ga]Ga-DOTA-TATE, especially in liver metastases.

#### 2.1.3 [^68^Ga]Ga-NODAGA-JR11

Fani et al. determined the effect of different radiometals and chelators on radiopharmaceutical binding to SSTR-positive NETs (16). Based on their research, [^68^Ga]Ga-NODAGA-JR11 demonstrated a lower half-maximum inhibitory concentration (IC_50_) than [^68^Ga]Ga-DOTA-JR11, suggesting a better tumor binding rate and imaging sensitivity. In 2023, Lin et al. completed a clinical trial with 48 patients comparing [^68^Ga]Ga-DOTA-TATE to [^68^Ga]Ga-NODAGA-JR11 to determine biodistribution, lesion uptake, sensitivity, and safety (17). 673 liver lesions were detected with [^68^Ga]Ga-NODAGA-JR11 compared to 584 with [^68^Ga]Ga-DOTA-TATE, causing an imaging sensitivity of 91.7% and 77.2%, respectively.

#### 2.1.4 [^68^Ga]Ga-DOTA-LM3

A phase 2 trial was completed by Zhu et al. to compare [^68^Ga]Ga-DOTA-LM3 and [^68^Ga]Ga-DOTA-TATE (18). Biodistribution was compared, and lower [^68^Ga]Ga-DOTA-LM3 uptake was demonstrated in all studied organs except the lungs and the blood pool. [^68^Ga]Ga-DOTA-LM3 detected 447 lesions while [^68^Ga]Ga-DOTA-TATE only detected 372. Both radiopharmaceuticals detected statistically equivalent primary tumors, bone metastases, and lymph node lesions, but more liver metastases were imaged using [^68^Ga]Ga-DOTA-LM3 compared to [^68^Ga]Ga-DOTA-TATE. With a similar tumor uptake and decreased biodistribution in healthy tissue, [^68^Ga]Ga-DOTA-LM3 demonstrated better TBRs and image contrast than [^68^Ga]Ga-DOTA-TATE (TBR = 5.2 and 2.1, respectively).

#### 2.1.5 [^68^Ga]Ga-NODAGA-LM3

In 2021, Zhu et al. completed a phase 1 trial comparing the tumor uptake, biodistribution, and effective dose of [^68^Ga]Ga-DOTA-LM3 and [^68^Ga]Ga-NODAGA-LM3 in 8 patients (19). Maximum tumor uptake was reached within the first 30 min and plateaued at an SUV_max_ of 45.3 ± 29.3. The highest absorbed dose in healthy tissue occurred in the urinary bladder wall at 0.202 mGy/MBq demonstrating renal excretion of [^68^Ga]Ga-DOTA-LM3. The overall average effective dose to patients was 0.026 ± 0.003 mSv/MBq, comparable to [^68^Ga]Ga-DOTA-JR11 and [^68^Ga]Ga-NODAGA-JR11.

**Figure 3:**
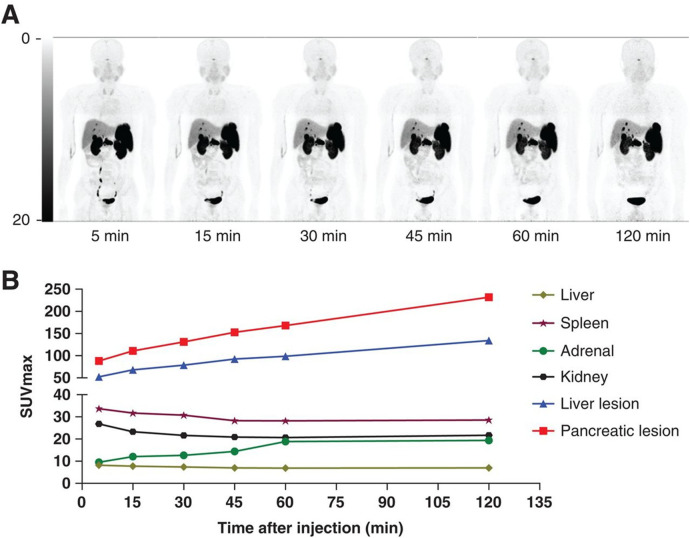
**(A)** Biodistribution of [^68^Ga]Ga-NODAGA-LM3 at 5, 15, 30, 45, 60, and 120 min after injection. **(B)** SUV_max_–time curves (19).

Zhu et al. progressed to a phase 2 clinical trial to compare [^68^Ga]Ga-NODAGA-LM3 and [^68^Ga]Ga-DOTA-TATE (18). In the study, [^68^Ga]Ga-NODAGA-LM3 demonstrated lower uptake in the liver, spleen, thyroid, stomach, and small intestine compared to [^68^Ga]Ga-DOTA-TATE, but higher uptake in the lungs and blood pool. [^68^Ga]Ga-NODAGA-LM3 detected more lesions than [^68^Ga]Ga-DOTA-TATE with 395 and 339 lesions detected, respectively. No statistically significant difference was observed for primary tumors, bone metastases, and lymph node metastases comparing [^68^Ga]Ga-NODAGA-LM3 and [^68^Ga]Ga-DOTA-TATE. [^68^Ga]Ga-NODAGA-LM3 demonstrated statistically higher tumor uptake than [^68^Ga]Ga-DOTA-TATE showing a median SUV_max_ of 29.1 and 21.6, respectively. Figure 3 compares the SUV_max_ in healthy organs of interest to pancreatic and liver lesions to demonstrate the high TBR. Both novel radiopharmaceuticals demonstrated higher diagnostic efficacy, with 61% of patients having more lesions identified with [^68^Ga]Ga-NODAGA-LM3% and 44% of patients having more lesions identified with [^68^Ga]Ga-DOTA-LM3 than [68 Ga]Ga-DOTA-TATE. Both novel radiopharmaceuticals using LM3 detected more liver metastases than [^68^Ga]Ga-DOTA-TATE and did not exhibit decreased uptake in bone metastases, unlike [^68^Ga]Ga-DOTA-JR11.

Zhu et al. completed a trial in 2022 comparing the SUV_max_ values of [^68^Ga]Ga-NODAGA-LM3 to [^68^Ga]Ga-DOTA-LM3 and [^68^Ga]Ga-NODAGA-JR11. The results of the study are shown in Table 2 (20).

**Table 2:** Comparison of median SUV_max_ for novel ^68^Ga and ^18^F radiopharmaceuticals.

Radiopharmaceutical	Median SUV_max_	Citation
[^68^Ga]Ga-DOTA-JR11	21 ± 21	(14)
[^68^Ga]Ga-DOTA-LM3	19.8 ± 17.2	(20)
[^68^Ga]Ga-NODAGA-JR11	25.0 ± 20.0	(20)
[^68^Ga]Ga-NODAGA-LM3	35.3 ± 28.8	(18–20)
[^68^Ga]Ga-NOTA-3P-TATE-RGD	27.2 ± 13.6	(21)
[^18^F]FAlF-NOTA-TOC	12.3 ± 6.5	(22)
[^18^F]F-SiFAlin-TATE	18.8 ± 8.4 (liver lesions)	(27)
[^18^F]FET-βAG-TOCA	19.2 ± 21.1	(30)
[^18^F]AlF-NOTA-LM3	10.2–49.6	(31)

#### 2.1.6 [^68^Ga]Ga-NOTA-3P-TATE-RGD

[^68^Ga]Ga-NOTA-3P-TATE-RGD is a dual SSTR2 and integrin alpha(v)beta (3) targeting tracer. Jiang et al. completed a head-to-head comparison of [^68^Ga]Ga-NOTA-3P-TATE-RGD and [^68^Ga]Ga-DOTA-TATE for PET imaging of gastroenteropancreatic NETs (21). The trial found [^68^Ga]Ga-NOTA-3P-TATE-RGD and [^68^Ga]Ga-DOTA-TATE had similar uptake in primary, lymph node, and bone lesions, but [^68^Ga]Ga-NOTA-3P-TATE-RGD demonstrated significantly higher uptake in liver lesions with a higher tumor to background ratio in the liver.

### 2.2 Flourine-18 (^18^F) in focus

#### 2.2.1 [^18^F]AlF-NOTA-TOC

The first comparison, involving 12 patients (6 healthy and 6 with diagnosed NETs), was conducted in 2020 by Pauwels et al. This study marked the first comparison of [^18^F]AlF-NOTA-TOC and [^68^Ga]Ga-DOTA-TATE based on biodistribution and lesion detection rates (22). Before the comparison, the pharmacokinetics, dosimetry, and safety of [^18^F]AlF-NOTA-TOC were determined. The [^18^F]AlF-NOTA-TOC mean effective dose was 22.4 ± 4.4 μSv/MBq, with the highest dose going to the spleen, bladder wall, and kidneys from renal excretion. No significant difference in tumor to background ratio was determined comparing [^18^F]AlF-NOTA-TOC and [^68^Ga]Ga-DOTA-TATE, but [^68^Ga]Ga-DOTA-TATE had higher uptake in the salivary glands. Comparing peak uptake, [^18^F]AlF-NOTA-TOC detected 91.7% of lesions while [^68^Ga]Ga-DOTA-TATE detected 86%. A trial conducted by Haeger et al. in 2023 compared the biodistribution and tumor uptake of [^18^F]AlF-NOTA-TOC and [^68^Ga]Ga-DOTA-TATE in the Latin American population (23). The study found [^18^F]AlF-NOTA-TOC was non-inferior to [^68^Ga]Ga-DOTA-TATE.

**Figure 4:**
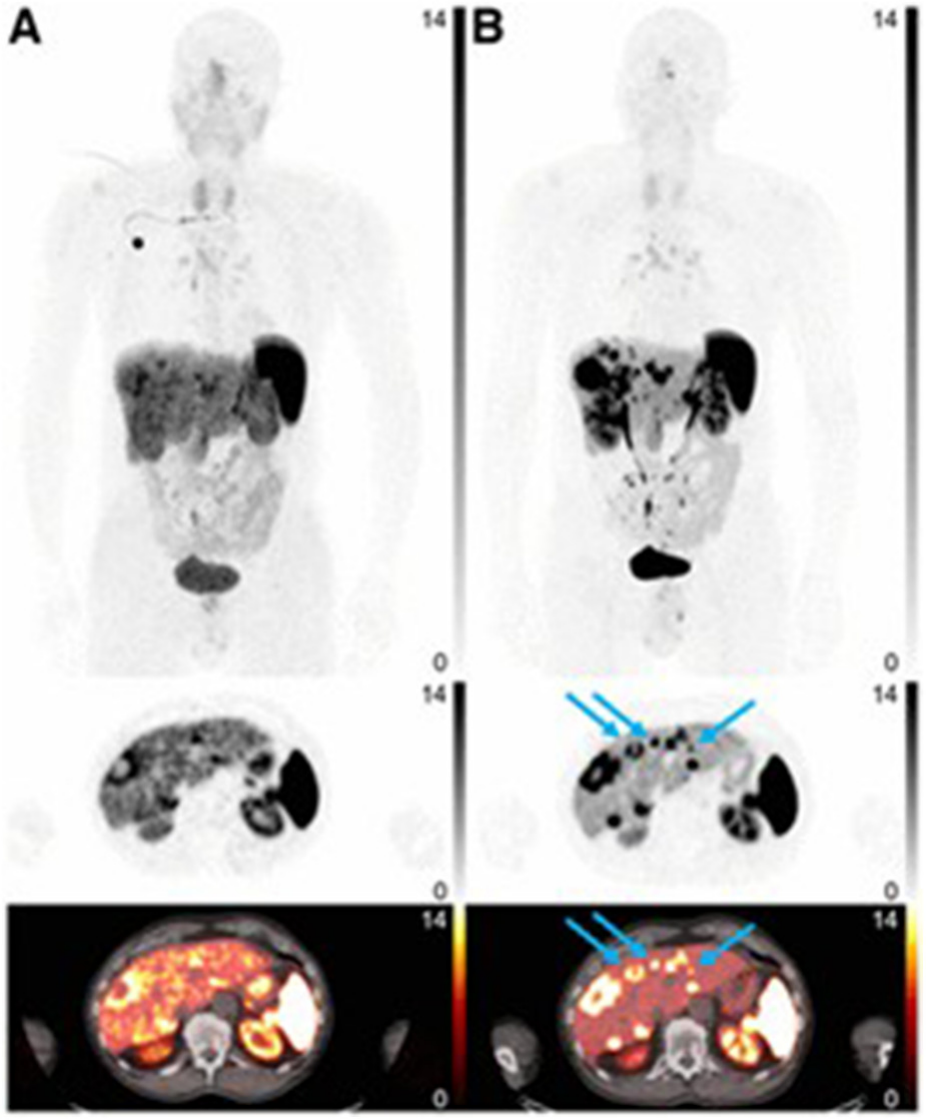
[^68^Ga]Ga-DOTATATE **(A)** and [^18^F]AlF-NOTA-TOC **(B)** images. Arrows indicate missed liver lesions using [^68^Ga]Ga-DOATATE. Intensity scale bars indicate SUVs. (25).

Pauwels et al. and Boekxstaens et al. completed follow-up multicenter studies comparing [^18^F]AlF-NOTA-TOC and [^68^Ga]Ga-DOTA-TATE/NOC (24, 25). The mean detection rate was significantly greater for [^18^F]AlF-NOTA-TOC than [^68^Ga]Ga-DOTA-TATE/NOC: 99.1–91.1% vs. 91.4–75.3%. The differential detection rate was above zero for [^18^F]AlF-NOTA-TOC in most organs except bone lesions, where the detection rate was not significantly different which can be seen in Figure 4. Based on the data reported by Pauwels et al. and Boeckxstaens et al., [^18^F]AlF-NOTA-TOC showed superiority over [^68^Ga]Ga-DOTA-TATE/NOC for imaging of NET patients. A trial completed by Leupe et al. in 2024 compared [^18^F]AlF-NOTA-TOC and [^68^Ga]Ga-DOTA-TATE/NOC in 75 patients to determine clinical impact on tumor, nodes, and metastasis (TNM) staging and clinical management (26). In 86.7% of patients, no difference in TNM staging was seen between [^18^F]AlF-NOTA-TOC and [^68^Ga]Ga-DOTA-TATE/NOC, but [^18^F]AlF-NOTA-TOC detected more lesions in 9.3% of patients, causing increased TNM staging. Even with the change in TNM staging, the large majority of patients saw no significant impact on TNM staging or clinical management compared to [^68^Ga]Ga-DOTA-TATE/NOC, demonstrating the noninferiority of [^18^F]AlF-NOTA-TOC.

#### 2.2.2 [^18^F]SiFAlin-TATE

In 2020, Ilhan et al. presented the first in-human trial for [^18^F]SiFAlin-TATE to determine biodistribution, tumor uptake, and compare image quality to [^68^Ga]Ga-DOTA-TOC (27). The study found [^18^F]SiFAlin-TATE had higher uptake in the adrenal glands, liver, spleen, myocardium, muscle, and bone than [^68^Ga]Ga-DOTA-TOC. [^18^F]SiFAlin-TATE demonstrated higher uptake in most tumor lesions with a significantly higher SUV_max_ in liver, lymph node, and bone lesions, with the only exception being lower uptake in lung lesions. Image quality was compared using blind readers, and it was found that readers preferred [^18^F]SiFAlin-TATE in 21% of scans and [^68^Ga]Ga-DOTA-TOC in 28% of scans. Based on the study, [^18^F]SiFAlin-TATE demonstrated comparable results to [^68^Ga]Ga-DOTA-TOC with partly higher TBR, indicating the need for further clinical investigation. A following trial completed in 2021 by Beyer et al. determined dosimetry and optimal scanning time for 8 patients (28). The total effective dose was 0.015 ± 0.004 mSv/MBq, which is a similar and slightly lower effective dose compared to [^68^Ga]Ga-DOTA-TATE/TOC, showing promise in the clinic. Eschbach et al. compared TBR and SUV_max_ values for 77 patients with (40 patients) or without (37 patients) SSA therapy before imaging (29). The study found a decrease in the SUV_max_ in normal liver and spleen tissue without any significant impact on TBR contrast, demonstrating no effect of SSA therapy on image quality using [^18^F]SiFAlin-TATE.

#### 2.2.3 [^18^F]FET-βAG-TATE

In 2024, Dubash et al. completed a phase 2 clinical trial imaging 45 patients to compare [^18^F]FET-βAG-TOCA and [^68^Ga]Ga-DOTA-TATE (30). The median SUV_max_ was significantly different in the liver, where the [^18^F]FET-βAG-TATE TBR was significantly lower. The study demonstrated that [^18^F]FET-βAG-TATE is not inferior to [^68^Ga]Ga-DOTA-TATE.

#### 2.2.4 [^18^F]AlF-NOTA-LM3

Liu et al. completed a clinical trial in 2024 to determine the biodistribution and dosimetry of [^18^F]AlF-NOTA-LM3 and compare tumor uptake and TBR of [^18^F]AlF-NOTA-LM3 and [^68^Ga]Ga-DOTA-TATE (31). In the trial, 21 patients were divided into two groups: an 8-patient group to test biodistribution and dosimetry, and a 13-patient group for radiopharmaceutical comparison. [^18^F]AlF-NOTA-LM3 was found to have significantly lower uptake in healthy organs except the blood pool, lungs, and gallbladder compared to [^68^Ga]Ga-DOTA-TATE. [^18^F]AlF-NOTA-LM3 detected more liver and lymph node lesions than [^68^Ga]Ga-DOTA-TATE and was comparable in detecting primary and bone lesions. With a lower uptake in most organs, [^18^F]AlF-NOTA-LM3 demonstrated a higher TBR and detected significantly smaller lesions than [^68^Ga]Ga-DOTA-TATE (0.54 ± 0.15 vs. 1.01 ± 0.49 for liver lesions). [^18^F]AlF-NOTA-LM3 demonstrated favorable biodistribution, higher TBR, and better spatial resolution than [^68^Ga]Ga-DOTA-TATE, indicating feasibility for routine clinical use of [^18^F]AlF-NOTA-LM3. Another trial, completed in 2024 by Zhang et al., compared SUV_max_ and TBR for free-breathing, respiratory-gated, and breath-hold PET scans using [^18^F]AlF-NOTA-LM3 (32). The SUV_max_ are compared for novel ^68^Ga and ^18^F radiopharmaceuticals in Table 2 where ^18^F labelled tracers show comparable but slightly lower SUV_max_ compared to the ^68^Ga counterparts.

#### 2.2.5 [^18^F]AlF-NOTA-JR11

Xie et al. completed a preclinical and pilot clinical study comparing [^18^F]AlF-NOTA-JR11 and [^68^Ga]Ga-DOTA-TATE in 2021 (33). The pilot clinical trial included 10 patients diagnosed with neuroendocrine neoplasms, comparing the biodistribution and lesion detection capabilities. [^18^F]AlF-NOTA-JR11 uptake was lower than [^68^Ga]Ga-DOTA-TATE in healthy organs except the blood pool and lungs. Due to the lower liver uptake, the TBR for liver metastases was greater for [^18^F]AlF-NOTA-JR11, potentially causing 67 more liver lesions to be detected with [^18^F]AlF-NOTA-JR11 compared to [^68^Ga]Ga-DOTA-TATE. Xie et al. demonstrated the superiority of [^18^F]AlF-NOTA-JR11 over [^68^Ga]Ga-DOTA-TATE for imaging of lesions in the digestive system.

### 2.3 Technetium-99 m (^99m^Tc): A cornerstone in nuclear medicine

#### 2.3.1 [^99m^Tc]Tc-EDDA/HYNIC-TOC

[^99m^Tc]Tc-EDDA/HYNIC-TOC (Tektrotyd® POLATOM, Poland) was first tested in clinical trials in 2000 by Decristoforo et al. and received marketing authorization in Europe for diagnostic imaging of GEP-NETs in later years (34, 35). The clinical trials completed since 2020 have shared a common goal of determining the sensitivity, specificity, accuracy, and SUV_max_, of [^99m^Tc]Tc-EDDA/HYNIC-TOC.

Three trials have been completed since 2020 to test the sensitivity, specificity, and accuracy of [^99m^Tc]Tc-EDDA/HYNIC-TOC. In 2022, Gerghe et al. completed a clinical trial with 173 patients and found a sensitivity of 90.5%, a specificity of 71.9%, and an accuracy of 84.3% (36). In 2023, Saponjski et al. compared [^99m^Tc]Tc-EDDA/HYNIC-TOC and [^18^F]F-FDG sensitivity, specificity, and accuracy in 90 patients (37). They found [^99m^Tc]Tc-EDDA/HYNIC-TOC and [^18^F]F-FDG demonstrated similar sensitivity (83.6% vs. 92.0%) and accuracy (83.3% vs. 87.8%) but [^99m^Tc]Tc-EDDA/HYNIC-TOC had significantly better specificity than [^18^F]F-FDG (82.6% vs. 66.7%). The study results demonstrated [^99m^Tc]Tc-EDDA/HYNIC-TOC and [^18^F]F-FDG are comparable and can serve as useful complementary tools for imaging of patients with NETs. Additionally, in 2023, Moriguchi-Jeckel et al. compared [^99m^Tc]Tc-EDDA/HYNIC-TOC to [^111^In]In-DTPA-TOC in 9 patients (38). In the study [^99m^Tc]Tc-EDDA/HYNIC-TOC demonstrated better sensitivity in any image (93.7% vs. 74.8%) and in liver-specific images (97.8 vs. 85.1%) compared to [^111^In]In-DTPA-TOC. The specificity for both radiopharmaceuticals was equivalent, mainly caused by the low number of patients enrolled in the study. Moriguchi-Jeckel et al. showed [^99m^Tc]Tc-EDDA/HYNIC-TOC was an alternative to [^111^In]In-DTPA-TOC and may serve as a better imaging agent for liver metastases. While the three studies measured differing values for the sensitivity, specificity, and accuracy of [^99m^Tc]Tc-EDDA/HYNIC-TOC, all three studies demonstrated the potential for [^99m^Tc]Tc-EDDA/HYNIC-TOC to be used in the diagnostic imaging of patients with NETs.

The SUV_max_ was determined for [^99m^Tc]Tc-EDDA/HYNIC-TOC in four papers ranging from 2022–2024. In 2022, Piwowarska-Bilska et al. completed a trial with 42 patients to determine SUV_bw max_ (SUV_max_ values normalized to body weight) using an optimized method for estimating SUV_max_ (39). They found the SUV_bw max_ of the spleen was 39.3 ± 13.9 g/ml, the liver was 12.6 ± 2.7 g/ml, and liver lesions were 37.6 ± 27.1 g/ml showing a good tumor-to-background ratio in the liver. Gherghe et al. conducted another study in 2023 to investigate whether [^99m^Tc]Tc-EDDA/HYNIC-TOC SUV_max_ values could be used to assess treatment and prognosis (40). The study contained 14 patients and found SUV_max_ for liver lesions was 12.44 ± 7.76, for lymph node metastases was 11.98 ± 10.45, and for bone metastases was 5.90 ± 3.68 compared to 3.34 ± 0.93, 8.01 ± 5.31, and 0.66 ± 0.37 in healthy liver, lymph node, and bone tissue respectively. A trial completed by Gemmell et al. in 2023 compared SUV_max_ values for patients actively receiving long acting release somatostatin analogue (LAR-SSA) treatments to patients not receiving LAR-SSA treatments during the study (41). The study contained a total of 177 patients where 55 were receiving LAR-SSA therapy. The SUV_max_ value in nodal metastases was 19.2 ± 13.0 for patients receiving LAR-SSA and 17.4 ± 11.5 for patients without. In bone lesions, patients receiving LAR-SSA treatments had an SUV_max_ of 14.1 ± 13.5, and patients without treatment had 7.7 ± 8.0. The study found no significant effect on pathological uptake for primary and metastatic lesions apart from bone lesions while healthy organ uptake was significantly decreased with LAR-SSA therapy. This demonstrates the potential to improve tumor-to-background ratios and dosimetric results by continuing patient LAR-SSA treatments throughout imaging. The latest study completed in 2024 by Malarz et al. quantitively analyzed SUV_max_ values for metastatic bone lesions in 344 patients and found a SUV_bw max_ for bone lesions of 23.53 ± 28.70 whereas healthy bone was 2.54 ± 2.08 (42). With quantitative analysis of SUV_max_ values from SPECT/CT being feasible, [^99m^Tc]Tc-EDDA/HYNIC-TOC could serve as an alternative to PET/CT radiopharmaceuticals for monitoring and prognosis of NETs.

## 3 Targeted beta and alpha therapy agents

### 3.1 Lutetium-177 (^177^Lu): precision in targeted beta therapy

#### 3.1.1 [^177^Lu]Lu-DOTA-TATE

With the FDA approval of [^177^Lu]Lu-DOTA-TATE in 2018 and its subsequent approval for use in pediatric patients in 2024, the focus shifted toward [^177^Lu]Lu-DOTA-TATE dosimetry and combined therapy approaches. Studies completed in 2021 and 2022 found [^177^Lu]Lu-DOTA-TATE has a progression-free survival (PFS) of 12.5–16.92 months, with the 2024 LUTADOSE trial by Maccauro et al. presenting a larger median PFS of 42.1 months (43–45). Jacques et al. studied the impact of mRNA and miRNA expression on PFS before, during, and 6 months after therapy (46). The study found that low miRNA/mRNA expression after the first treatment correlated with patients developing progressive disease within 12 months after treatment and a decrease in lymphocyte count, indicating a potential for hematological toxicity development.

**Figure 5:**
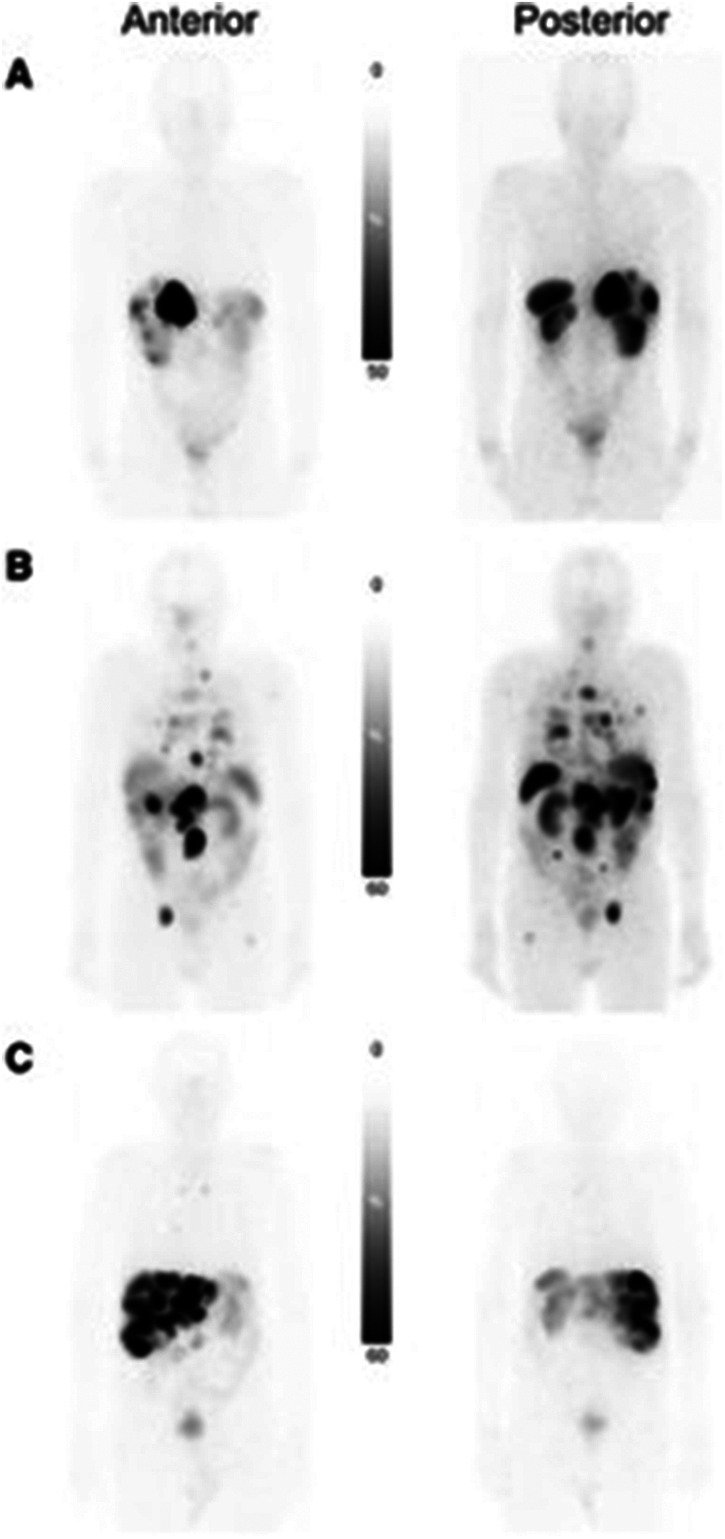
Planar scans of [^177^Lu]Lu-DOTA-TATE uptake in three patients with different tumor burden. **(A)** Patient with low tumor burden. **(B)** Patient with multiorgan involvement. **(C)** Patient with predominately liver-only metastatic disease with high liver tumor burden (48).

Dosimetry calculations using [^177^Lu]Lu-DOTA-TATE can be misleading because the radiopharmaceutical uptake and consequently absorbed dose from each cycle decreases (47). Palmese et al. compared dosimetry calculations throughout patient treatment and found absorbed dose calculations after the first cycle overestimated [^177^Lu]Lu-DOTA-TATE absorbed dose (47). Dosimetry calculations accounting for the first and last cycle can accurately model full cycle dose. With difficulty in accurately calculating patient absorbed dose, focus has been placed on absorbed dose calculations for organs at risk, where Bodei et al. demonstrated the organs with the highest uptake are the spleen (25.1 ± 23.8 Gy) and kidneys (19.4 ± 8.7 Gy) in planar scans shown in Figure 5 (48). Staanum et al., Spink et al., and Kapidzic et al. calculated patient kidney dose using varying methods and found a mean absorbed dose of 0.37–0.46 Gy/GBq (49–51). Due to the decrease in platelets, lymphocytes, and hemoglobin being correlated to red bone marrow absorbed dose, bone marrow is a limiting organ for [^177^Lu]Lu-DOTA-TATE, where Blakkisrud et al. and Hemmingsson et al. found a median absorbed dose in lumbar and thoracic vertebrae to be between 0.019 and 0.11 Gy/MBq and found a correlation between hematologic toxicity markers and red bone marrow dose (52, 53).

Lubberink et al. studied the stability of [^177^Lu]Lu-DOTA-TATE *in vivo* using 6 patients, where blood samples were collected at 30 min, 4 h, 24 h, and 96 h post-injection, and the fraction of intact radiopharmaceutical was only 23 ± 5% at 24 h (54).

[^177^Lu]Lu-DOTA-TATE FDA-approval was expanded to include adolescents ages 12–18 in April 2024. The FDA-approval was expanded based on results presented by the currently ongoing NETTER-P (NCT04711135) trial for adolescent patients and extrapolation of efficacy outcomes from the adult patient NETTER-1 trial. The NETTER-P trial reported safety and dosimetry results for 11 patients and found no significant safety markers or treatment related nephrotoxicity. The study projected a median dose of 21 Gy to kidneys and 0.76 Gy to red bone marrow which are believed to be within safety margins for adolescents (55).

#### 3.1.2 [^177^Lu]Lu-HA-DOTA-TATE

HA-DOTA-TATE is a conjugate of DOTA-TATE where the phenol group is replaced with an iodophenol group, which showed increased binding affinity for SSTR5 compared to DOTA-TATE while maintaining comparable SSTR1, 2, 3, and 4 binding affinity (56). Siebinga et al. determined the usability of [^68^Ga]Ga-HA-DOTA-TATE for individual predictive dosimetry of [^177^Lu]Lu-HA-DOTA-TATE and found a tumor relative prediction error of −40%–28% and kidney of −58%–82%, demonstrating the need for better predictive dosimetry imaging agents compared to ^68^Ga (57). Veerman et al. compared tumor uptake of [^177^Lu]Lu-HA-DOTA-TATE with and without long-acting octreotide or lanreotide therapy and found that LAR-SSA therapy did not affect tumor uptake, while healthy liver parenchyma and spleen uptake decreased (58).

#### 3.1.3 [^177^Lu]Lu-DOTA-EB-TATE

[^177^Lu]Lu-DOTA-EB-TATE contains an Evans blue motif, which uses endogenous albumin as a reversible carrier to increase the blood half-life and increase retention time within a tumor. Liu et al. completed a dose escalation study to compare [^177^Lu]Lu-DOTA-EB-TATE to [^177^Lu]Lu-DOTA-TATE for patient response (59). The study found that patients treated with [^177^Lu]Lu-DOTA-EB-TATE had a 50% partial response rate, whereas only 16.7% of patients treated with [^177^Lu]Lu-DOTA-TATE demonstrated a partial response. Jiang et al. completed a trial comparing kidney dose with and without an amino acid cocktail for [^177^Lu]Lu-DOTA-EB-TATE and found a statistically insignificant increase in kidney dose without an amino acid cocktail and no change in renal toxicity (60).

#### 3.1.4 [^177^Lu]Lu-DOTA-JR11

[^177^Lu]Lu-satoreotide tetraxetan [(^177^Lu)Lu-DOTA-JR11] has shown promise as an alternative therapy agent to [^177^Lu]Lu-DOTA-TATE. Wild et al. completed a phase I/II clinical trial to compare patients receiving 6.0 GBq to 4.5 GBq per cycle for three cycles and found that 4.5 GBq per cycle was recommended to prevent excess bone marrow dose (61). The decrease to 4.5 GBq/cycle with three cycles achieved comparable results to [^177^Lu]Lu-DOTA-TATE using the recommended 7.4 GBq/cycle. Schürrle et al. also completed a phase I/II clinical trial to evaluate dosimetry and pharmacokinetics of [^177^Lu]Lu-DOTA-JR11 in 40 patients (62). The study found three cycles of 4.5 GBq/300 μg were tolerable to the kidneys, liver, and spleen, with absorbed dose coefficients of 0.9 Gy/GBq, 0.2 Gy/GBq, and 0.8 Gy/GBq, respectively.

### 3.2 Terbium-161 (^161^Tb): the therapeutic hybrid

#### 3.2.1 [^161^Tb]Tb-DOTA-TOC

^161^Tb has shown improved therapeutic effectiveness compared to ^177^Lu due to its dual targeted beta therapy and targeted Auger electron therapy modalities which may lead to increasing the cytotoxicity (63). The emission of several Auger and conversion electrons per decay increases ^161^Tb cytotoxicity. Similar to ^177^Lu decay, which emits gammas in the SPECT imaging range, ^161^Tb can also decays with the emission of gammas allowing dosimetry calculations based on SPECT imaging. In 2021, Baum et al. completed the first and only in-human trial for [^161^Tb]Tb-DOTA-TOC to determine the SPECT resolution of ^161^Tb for imaging of multiple paraganglioma and pancreatic neuroendocrine neoplasm metastases (63). Patients were compared with doses of 596 and 1300 MBq, and patients underwent five whole-body planar images and two SPECT/CT images. Both doses demonstrated high-resolution imaging and could detect all lesions seen by [^68^Ga]Ga-DOTA-TATE imaging for both injection activities. The 596 MBq dose demonstrated similar dosimetry results to [^177^Lu]Lu-DOTA-TATE while the 1,300 MBq dose demonstrated increased dose to the kidneys.

#### 3.2.2 [^161^Tb]Tb-DOTA-LM3

In 2024, Fricke et al. completed the first and only human administration of [^161^Tb]Tb-DOTA-LM3, treating and imaging a patient with metastatic ileal NETs (64). The patient received a single 1 GBq injection, demonstrating favorable biodistribution, tumor absorbed dose, and SPECT/CT imaging capabilities. The study found good image quality at 168 h post-injection with a therapeutic level mean tumor absorbed dose of 28 Gy/GBq. At the same time, organs at risk stayed below constraint limits at 0.31, 3.33, and 6.86 Gy/GBq for bone marrow, kidney, and spleen, respectively. The patient saw a decrease in tumor marker chromogranin A levels, indicating therapeutic impact.

### 3.3 Lead-212 (^212^Pb): innovation and future directions

#### 3.3.1 [^212^Pb]Pb-DOTAM-TATE

Delpassand et al. completed the first in-human dose-escalation clinical trial in 2022, involving 22 patients (65). Of the patients, 10 received up to 2.50 MBq/kg/cycle, which is the recommended phase 2 dose regimen, with the main adverse effects being alopecia and nausea. The 10 patients exhibited an overall response rate of 80%, demonstrating the clinical potential of [^212^Pb]Pb-DOTATATE. Based on the results presented in Delpassand et al.'s trial, the FDA has granted [^212^Pb]Pb-DOTATATE (AlphaMedix) breakthrough therapy designation for the treatment of neuroendocrine tumors and has prompted a currently active, not recruiting Phase 2 trial as of May 2024 (clinicaltrials.gov, NCT05153772).

#### 3.3.2 [^212^Pb]VMT-a-NET

[^212^Pb]VMT-a-NET is a lead based radiopharmaceutical utilizing a novel chelator for conjugation to octreotide. The radiopharmaceutical is currently in its first open-label, multi-center, dose-escalation/dose expansion, phase I/IIa clinical trial. The trial is currently recruiting patients as of March 2025 (Clinicaltrial.gov NCT05636618) (66).

### 3.4 Copper-67 (^67^Cu): emerging applications in targeted radiotherapy

#### 3.4.1 [^67^Cu]Cu-MeCOSAR-TATE

In 2023, Bailey et al. completed the first clinical trial evaluating the theragnostic pair of [^64/67^Cu]Cu-MeCOSAR-TATE [(^64/67^Cu)Cu-sartate] (67). Three patients received 4 cycles and SPECT and PET images were compared to ensure identical targeting indicative of the matched pair theragnostic. No serious adverse events were observed during the treatment and patients received a mean effective dose of 7.62 × 10^−2^ mSv/MBq. The trial did not report estimated absorbed dose to therapeutic targets, dose-response relationships, or efficacy due to limited SPECT spatial resolution of small lesions. As of July 2024, ([^64/67^Cu]Cu-sartate is in a recruiting phase I/II trial for treatment of pediatric patients with high-risk, relapsed, refractory neuroblastoma (Clinicaltrial.gov NCT04023331).

### 3.5 Actinium-225 (^225^Ac): advancement in targeted alpha therapy

#### 3.5.1 [^225^Ac]Ac-DOTA-TATE

The alpha-emitting [^225^Ac]Ac-DOTA-TATE has been the main clinical focus of the new decade. The long-term effects of [^225^Ac]Ac-DOTA-TATE were reported in a 2023 study by Ballal et al. containing 91 adults with GEP-NETs (68). Patients received capecitabine therapy during days 0 through 14 of the [^225^Ac]Ac-DOTA-TATE treatment cycle. [^225^Ac]Ac-DOTA-TATE therapy was found to improve the OS, including in patients who had previously demonstrated resistance to ^177^Lu-PRRT, with transient and acceptable adverse effects. In addition to GEP-NETs, the efficacy and safety of [^225^Ac]Ac-DOTA-TATE for targeted alpha therapy in metastatic paragangliomas have been investigated in a pilot study involving 9 patients treated with [^225^Ac]Ac-DOTA-TATE and concomitant capecitabine at 8-weekly intervals, resulting in a cumulative administered activity of 74 MBq. Following treatment, partial responses, stable disease, and disease progression were observed in 50%, 37.5%, and 12.5% of patients, respectively. A clear benefit was noted in patients refractory to previous ^177^Lu-PRRT (69). ACTION-1 (NCT05477576) is an ongoing clinical trial for patients with advanced, well-differentiated, SSTR + GEP-NETS that are progressing after ^177^Lu-PRRT therapy.

## 4 Conclusions

Significant advancements and developments have occurred in the clinical implementation of SSTR-targeted radiopharmaceuticals for diagnosing and treating NETs over the past five years. Radiolabeled SSTR antagonists—such as [^68^Ga]Ga-DOTA-JR11 and [^68^Ga]Ga-DOTA-LM3—have marked a turning point in PET imaging. While traditional agonists, such as [^68^Ga]Ga-DOTA-TATE and [^68^Ga]Ga-DOTA-TOC, rely on receptor internalization, antagonists offer enhanced imaging sensitivity by binding to a broader range of receptor conformations, including inactive sites. This results in improved TBRs, particularly beneficial in detecting hepatic metastases where diagnostic accuracy has historically been challenging due to healthy liver tissue uptake. These improvements in image resolution and contrast directly support earlier and more confident disease localization, enabling more informed therapeutic decisions. Similarly, the emergence of ^18^F and 64Cu-labeled complexes, such as [^18^F]AlF-NOTA-TOC and FDA-approved [^64^Cu]Cu-DOTA-TATE, offers alternatives to ^68^Ga-labeled tracers with extended half-life and cyclotron-based production, increased imaging flexibility, and providing increased patient access for centers without a ^68^Ga generator. Moreover, clinical trials consistently report either comparable or improved sensitivity and lesion detection rates using ^18^F-labeled compounds, further reinforcing their clinical viability.

On the therapeutic front, [^177^Lu]Lu-DOTA-TATE remains the current standard of care for PRRT, particularly in patients with well-differentiated, SSTR-positive NETs. Despite its FDA approval, studies continue highlighting the need for improved dosimetric approaches. The correlation between red bone marrow and kidney dose with hematologic and renal toxicities necessitates more refined, cycle-specific dosimetry to minimize the organ at risk dose. These findings underscore the importance of integrating quantitative imaging and individualized treatment planning to optimize patient outcomes. [^161^Tb]Tb-DOTA-TOC/LM3 has shown potential as an improved theragnostic agent compared to [^177^Lu]Lu-DOTA-TATE by leveraging the combined targeted beta therapy, targeted Auger electron therapy, and SPECT imaging modalities. With ^161^Tb demonstrating theragnostic efficacy in clinical trials, radionuclide production and separation chemistry have received increased attention to improve availability for increased patient access. With recent production advancements increasing availability, targeted beta therapy using ^67^Cu has gained momentum with [^67^Cu]Cu-sartate in beginning stage clinical trials.

Current therapeutic focus has been placed on targeted alpha therapy, which is emerging as a potential therapeutic modality for high-grade or treatment-refractory NETs. Targeted alpha therapy agents, such as [^225^Ac]Ac-DOTA-TATE, are being investigated due to the high linear energy transfer of alpha particles, which causes localized cytotoxicity while minimizing off-target damage. Early-phase clinical trials with [^225^Ac]Ac-DOTA-TATE have demonstrated tumor control in patients with prior progression who underwent [^177^Lu]Lu-DOTA-TATE therapy, with manageable toxicity profiles. While ^225^Ac has shown promise as a targeted alpha therapy agent, difficulties with availability and the long decay chain hinder clinical translatability. [^212^Pb]Pb-DOTA-TATE has earned FDA breakthrough therapy designation due to its potential ability to impact patient care by leveraging the decay of ^212^Pb and its daughter products for combined targeted beta and targeted alpha therapy.

Future research should prioritize the development of theragnostic pairs that couple diagnostic and therapeutic radiopharmaceuticals with matching pharmacokinetics and biodistribution profiles. Such pairings would enable improved biodistribution, disease localization, clearance rates, and predictive dosimetry before administering the therapeutic counterpart. Among emerging radiopharmaceuticals, ^161^Tb, ^67^Cu, and ^212^Pb stand out due to their potential for matched pair theragnostics utilizing ^1651^Tb, ^64^Cu, and ^203^Pb to tailor care to the individual patient. ^161^Tb and ^212^Pb have the potential to be more effective than ^177^Lu due to their unique nuclear decay characteristics which provide additional therapeutic Auger electrons and alpha particles alongside, respectively.

Despite advancements in adult patient therapy, gaps remain specifically in pediatric applications, dosimetry, and radionuclide availability. There is a notable lack in pediatric specific trials, with the only trials completed in the last 5 years pertaining solely to [^177^Lu]Lu-DOTA-TATE. The aforementioned pediatric trial led to the FDA-approval of [^177^Lu]Lu-DOTA-TATE in children and teenagers 12–18 years of age, six years after the initial approval for adult patients (55). There are currently no SSTR positive NET trials ongoing for novel radiopharmaceuticals in pediatric cases demonstrating the existing gap between adult and pediatric patient care. Dosimetry and radiobiological modeling requires significant advancement especially in terms of dosimetry for targeted alpha and targeted Auger electron therapy agents. These decay methods have significantly different LET than beta-emitters highlighting the need for novel dosimetry modeling methods as conventional dosimetry methods will not apply for other decay modes. Microdosimetry and individualized pharmacokinetic modeling are needed to avoid organ toxicity (70). Accurate dosimetry modelling is essential for the introduction of novel radiopharmaceuticals utilizing multimodal therapy such as ^161^Tb and ^212^Pb. For ^161^Tb, the micrometer range of the Auger electron is poorly modeled leading to patient dose being over or under estimated when using traditional MIRD based dosimetry calculations (71). ^212^Pb and ^225^Ac have high LET and long decay chains which complicate modelling. Dosimetry models cannot account for redistribution of daughter radionuclides and the associated dose to healthy tissue.

In summary, the ongoing evolution of radiolabeled SSTR-targeting agents has significantly enhanced the precision and efficacy of NET management. With promising developments in imaging contrast, dosimetry modeling, and targeted alpha therapy, future work will focus on patient-specific diagnostic and therapeutic approaches. Continued clinical trials and technological innovation will be essential to fully realize the potential of nuclear medicine in improving outcomes for patients with NETs.
